# Back from the Tip of the Nose

**DOI:** 10.1100/tsw.2001.26

**Published:** 2001-06-05

**Authors:** Giulio Cossu

**Affiliations:** Stem Cell Research Institute, H.S. Raffaele Milan and Dept. of Histology and Medical Embryology, II Medical School, University of Rome, La Sapienza, Italy

**Keywords:** neural (stem cell), lineage restriction

## Abstract

About 130 years ago, Giulio Bizozzero, then in Pavia, made a seminal observation [1]. He divided the tissues of the vertebrate body into three categories: those that divide constantly (labile), such as blood and skin, those that never divide, such as striated muscle and brain (perennial), and those that normally do not divide but can do so if injured (stable). As a consequence, diseases that perturb cell division, such as cancer, affect labile tissues, while degenerative diseases affect perennial tissues where repair is inefficient. Epithelia and blood possess a reservoir of cells that divide and maintain a progenitor pool throughout life (the stem cells) whereas striated muscle and brain were supposed not to contain stem cells. Furthermore, stem cells were supposed to generate only the cells of the tissue where they belong.

In recent years, things have changed radically. Several investigators have succeeded in isolating and expanding cells from the sub-ventricular zone of fetal and adult mouse brain. These cells can be grown *in vitro* indefinitely and yet maintain the capacity to generate all the cell types of the central nervous system, i.e., neurons, oligodendrocytes, and astrocytes. Therefore, these cells fulfill the criteria of stem cells and have been termed neural stem cells [2,3]. Within the last 3 years novel and unpredicted results have challenged the paradigm of lineage restriction of neural stem cells as well as of stem cells from other tissues. In 1998, Ferrari and colleagues showed that the bone marrow contains a transplantable progenitor that is capable of giving rise to new skeletal muscle [4]. The following year, Bjorson and colleagues showed that neural stem cells could reconstitute the hematopoietic system of the mouse upon bone marrow transplantation [5]. In the same year other reports demonstrated the existence of adult "stem" cells with broader, nonlineage-restricted multi-potentiality, residing in a variety of tissues, such as brain, muscle, and bone marrow[6-9].

Additional work published last year contributed to making the picture even more complex. Galli et al. reported that clonally derived adult neural stem cells can undergo myogenic differentiation, giving rise to multinucleated myotubes both *in vitro* and *in vivo* [10]. Thus, in addition to neurohematopoietic conversion, the repertoire of neuroectodermal-mesodermal transitions accessible to neural stem cells also includes a capacity for neuromyogenic conversion. It was suggested that local signaling may trigger a given pattern of differentiation in competent, multipotent stem cells. On the other hand, transition from mesoderm to neuroectoderm is also possible since purified hematopoietic stem cells can differentiate into skeletal muscle [8] or neurons and glia [11,12].

At this point the idea is in the air that each tissue contains stem cells that can give rise to virtually any tissue of the body; additional reports are now appearing at a weekly rate. Indeed, the press is flooded with reports, often unpublished, from companies claiming that almost any tissue or organ can be built starting from almost any cell. In a prophetic scene from the Woody Allen movie "Sleeper", a bomb destroys the car of the dictator, the tip of whose nose is the only piece rescued. Efficient doctors rebuild the whole dictator by amplifying and re-differentiating the few remaining cells. What was then (1973) the genial fantasy of an artist may soon correspond to the expectations of the general public.

There is clearly a great risk in raising excessive expectations. The case of gene therapy, which 10 years ago should have cured all genetic and acquired diseases in 5 years, is a good example of premature optimism and improper vulgarization.

In the case of stem cells, many questions remain to be answered, both in basic biology and in the perspective of clinical exploitation. For example, why do tissues such as the brain or the marrow contain stem cells that can give rise to other tissues such as skeletal muscle, normally not produced there? On a more general level, what is the developmental origin of these "stem" cells in post-embryonic tissues? We have proposed that pluripotent progenitor cells may be associated with the developing vasculature and delivered to all tissues during angiogenesis [13]. These progenitors would then differentiate according to the local microenvironment (i.e., satellite cells in muscle, osteoblasts in bone, etc.). Those that remain undifferentiated may also remain pluripotent because of their late and local commitment, and thus constitute the precursors of post-natal stem cells. This hypothesis still awaits experimental confirmation. At the level of tissue pathology, one may wonder whether metaplasia is a the consequence of errors in differentiation pathways of local stem cells. An important question to be answered is how locally produced signals recruit pluripotent cells to a given tissue fate and prevent improper differentiation into other cell type which would result in a teratoma-like structure. In this context, it will be crucial to identify the molecular switches that cause a pluripotent stem cell to adopt one or another cell fate. Whether or not these are the same molecular switches as those that dictate fate choices during embryogenesis, it will be necessary to activate them in every stem cell to obtain a homogeneous population of differentiated cells. Finally, it will be necessary to obtain solid evidence that the tissue newly built with pluripotent stem cells functions and endures much as the original tissue. These are difficult questions to answer and years may go by before we can re-derive functional organs from a combination of committed stem cells and appropriate matrices and biomaterials — one more good reason to recommend hard and solid work to the scientists involved and much more prudence to the press. Only by doing so can we hope that—now that the secret of pluripotency seems to be within reach—we will be able to produce the cornucopia of cells [14] necessary to ameliorate a large number of still untreatable diseases.

| | | |
|---|---|---|
| Received: | March | 5, 2001 |
| Accepted: | March | 26, 2001 |
| Published: | June | 5, 2001 |
